# Supplementary material for: Robust and accurate estimation of paralog-specific copy number for duplicated genes using whole-genome sequencing
Source: Nat Commun. 2022 Jun 9;13:3221. doi: 10.1038/s41467-022-30930-3 (PMC9184528; doi:10.1038/s41467-022-30930-3)
Supplement: Supplementary file 1 — Supplementary Information [file 41467_2022_30930_MOESM1_ESM.pdf]

Robust and accurate estimation of paralog-specific copy number for duplicated genes using whole-genome sequencing

Supplementary Information

Timofey Prodanov[1] and Vikas Bansal[2*]

[1] Bioinformatics and Systems Biology Graduate Program, University of California, San Diego, La Jolla CA 92093 USA.

[2] Department of Pediatrics, School of Medicine, University of California, San Diego, La Jolla CA 92093 USA.

[*] Corresponding author: vibansal@ucsd.edu

Contents

1 Supplementary Figures and Tables **2**
Supplementary Figure 1 2
Supplementary Figure 2 3
Supplementary Figure 3 4
Supplementary Figure 4 5
Supplementary Figure 5 6
Supplementary Figure 6 7
Supplementary Figure 7 8
Supplementary Figure 8 9
Supplementary Table 1 10
Supplementary Table 2 11

2 Supplementary Methods **12**
2.1 Creating homology table 12
2.2 Calculating background read depth 13
2.3 Re-mapping reads 14
2.4 Finding aggregate copy number profiles 14
2.4.1 Estimating number of *AggregateCN* states 14
2.4.2 HMM definition 14
2.4.3 Updating emission probabilities using scale parameters 15
2.4.4 Updating initial and transition probabilities 15
2.4.5 Speeding up HMM convergence 16
2.4.6 Log-likelihood convergence 16
2.4.7 Aggregate copy number quality 16
2.5 Estimating paralog-specific copy number using PSVs 17
2.5.1 EM algorithm 17
2.5.2 Information content of the PSVs 18
2.5.3 Selecting starting states for EM algorithm 19
2.6 Extending homology table to include an additional repeat copy for *OTOA* 19
2.7 Subsampling reads 20
2.8 Paralog-specific copy number validation using trios 20

1 Supplementary Figures and Tables

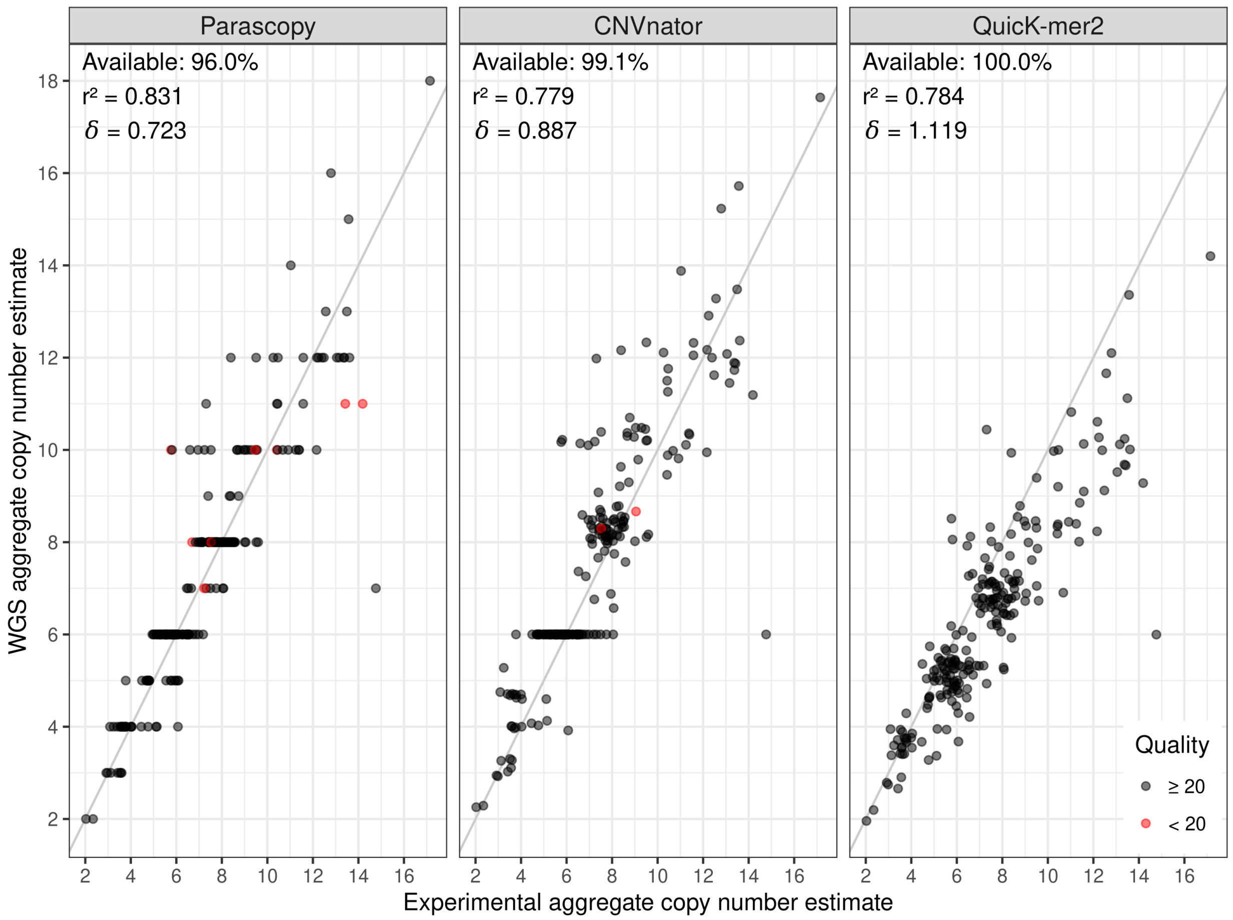

Supplementary Figure 1: **Comparison of experimental *AggregateCN* estimates for 225 1kGP samples at the AMY1A/B/C locus with Parascopy, CNVnator and QuicK-mer2 *AggregateCN* estimates.** The percentage of available *AggregateCN* estimates, Pearson correlation coefficient value (r^2) and average absolute error (δ) for each method are shown in the top left of each plot. Low quality copy number estimates are marked in red.

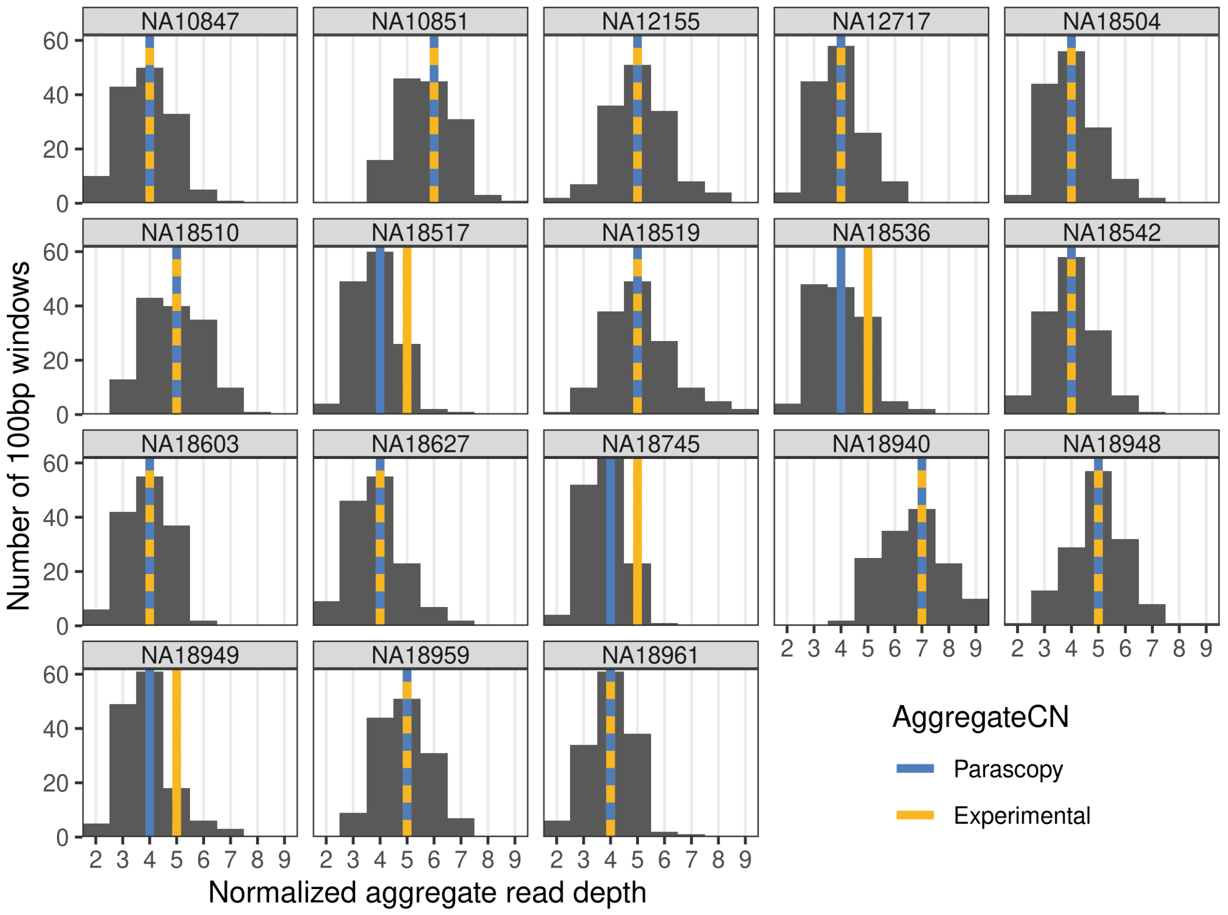

Supplementary Figure 2: **Comparison between Parascopy and experimental *AggregateCN* estimates for 18 1kGP samples.** Histograms show aggregate read depth distribution at 140 100 bp windows within NPY4R/2 duplication. Experimental *AggregateCN* values were obtained for 18 1kGP samples using ddPCR [1]. Parascopy *AggregateCN* estimates match with experimental values in 14 samples (shown with dashed blue-yellow lines). In the remaining 4 samples, Parascopy and ddPCR *AggregateCN* estimates are shown with separate blue and yellow vertical lines, respectively. The copy number estimates from CNVnator and QuicK-mer2 match Parascopy's estimates for all 4 samples in which Parascopy and experimental copy number estimates disagree.

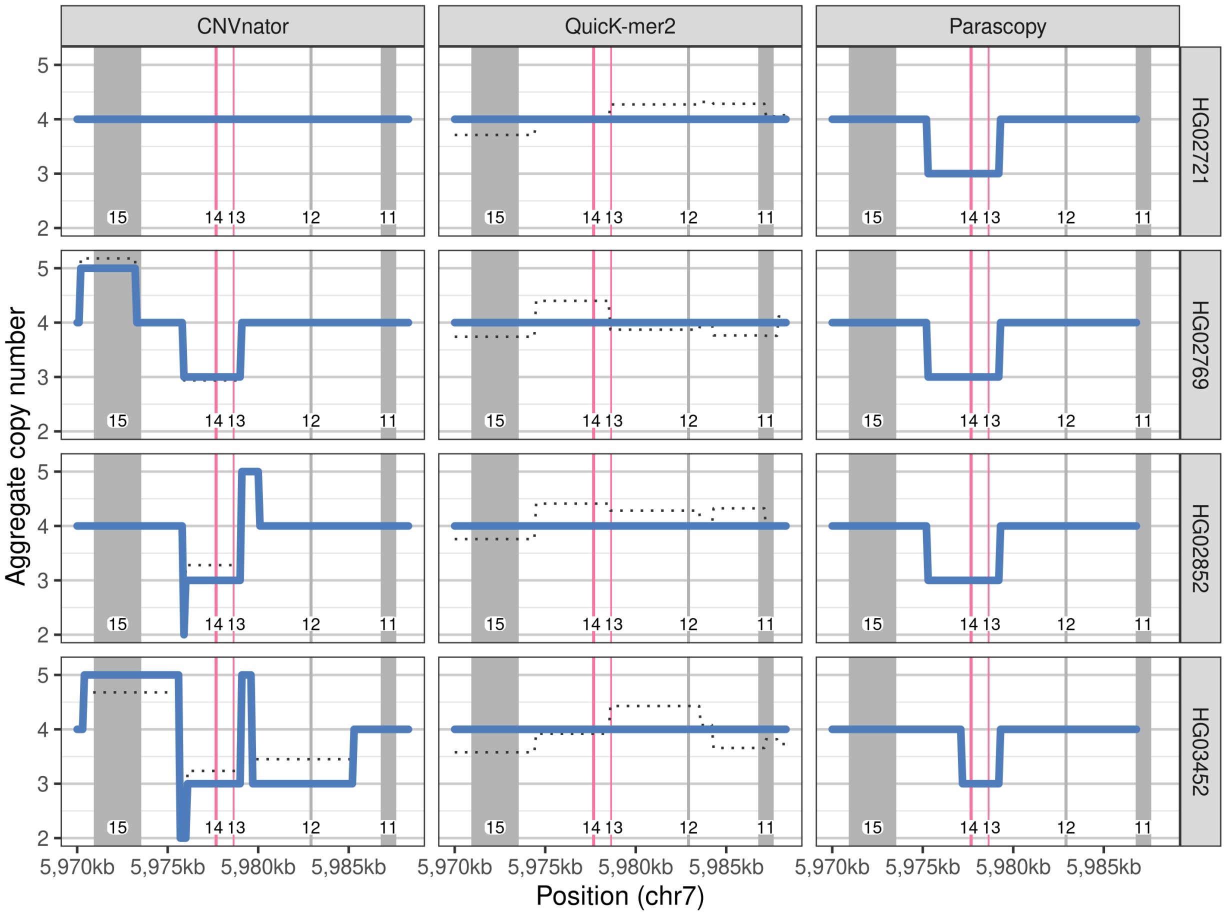

Supplementary Figure 3: **Comparison of CNVnator, QuicK-mer2 and Parascopy *AggregateCN* estimates for four samples at the PMS2 locus.** The four panels correspond to the four samples which were previously reported [2] to have a partial deletion within the PMS2CL pseudogene (overlapping PMS2 exons 13 and 14). The three vertical columns show copy number profiles for the three CNV-detection methods: CNVnator, QuicK-mer2 and Parascopy. Black dotted line shows fractional *AggregateCN* estimates, while the blue solid line shows integer *AggregateCN* estimates. Vertical gray and red rectangles display the duplicated PMS2 exons 11–15.

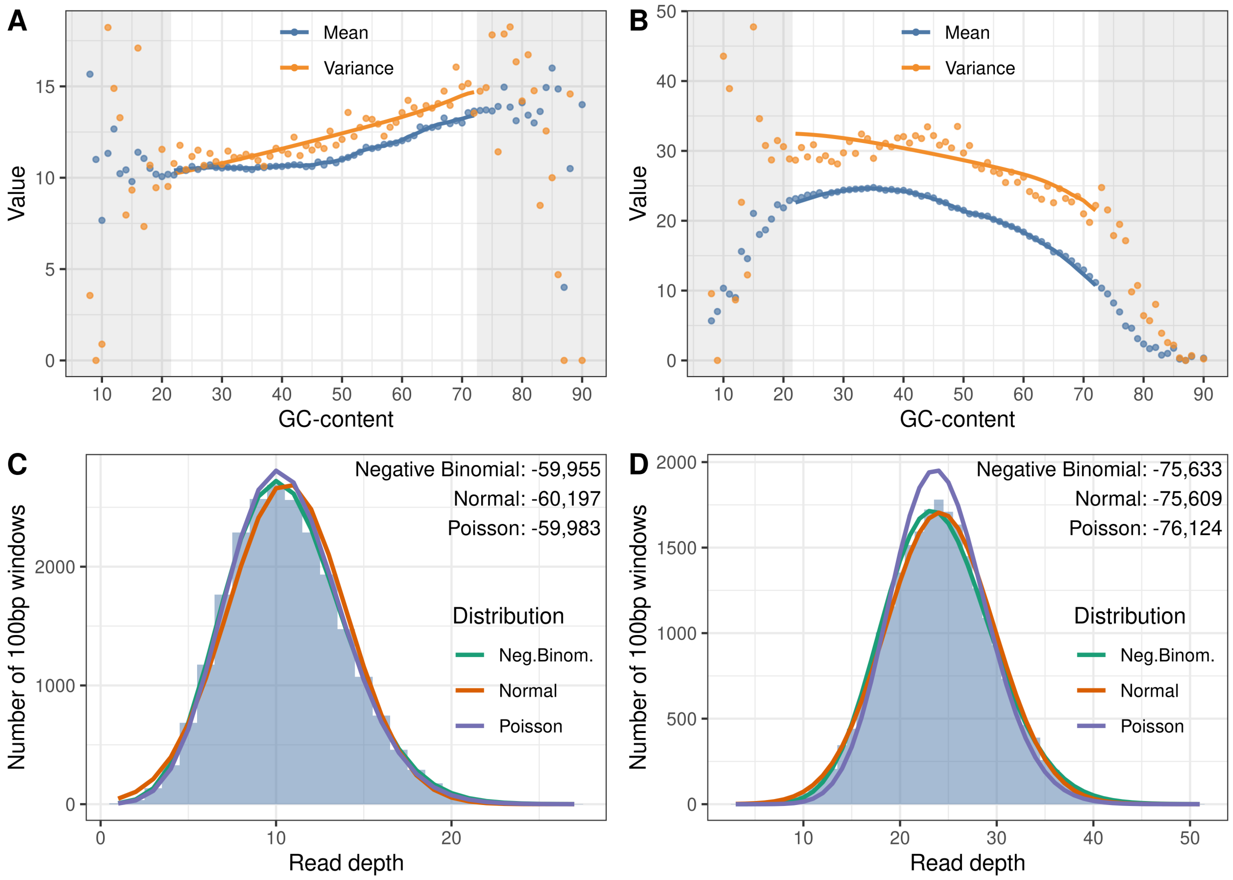

Supplementary Figure 4: **Estimation of mean read depth (and variance) using non-duplicated windows across the genome.** The mean and variance values across different GC-bins are shown for one sample with PCR-free WGS data (A) and PCR-based WGS data (B). Dots show empirical read depth mean and variance as a function of GC-content, while solid lines show smoothed mean and variance approximations, obtained using the LOWESS procedure [3,4] (not calculated in gray areas). Fit of various distributions (and corresponding log-likelihood values) to the read depth are shown for PCR-free data (C) and PCR-based data (D). For PCR-free WGS, both Negative Binomial and Poisson distributions give a similar fit of the read depth distribution while the Poisson has a significantly worse fit for PCR-based WGS.

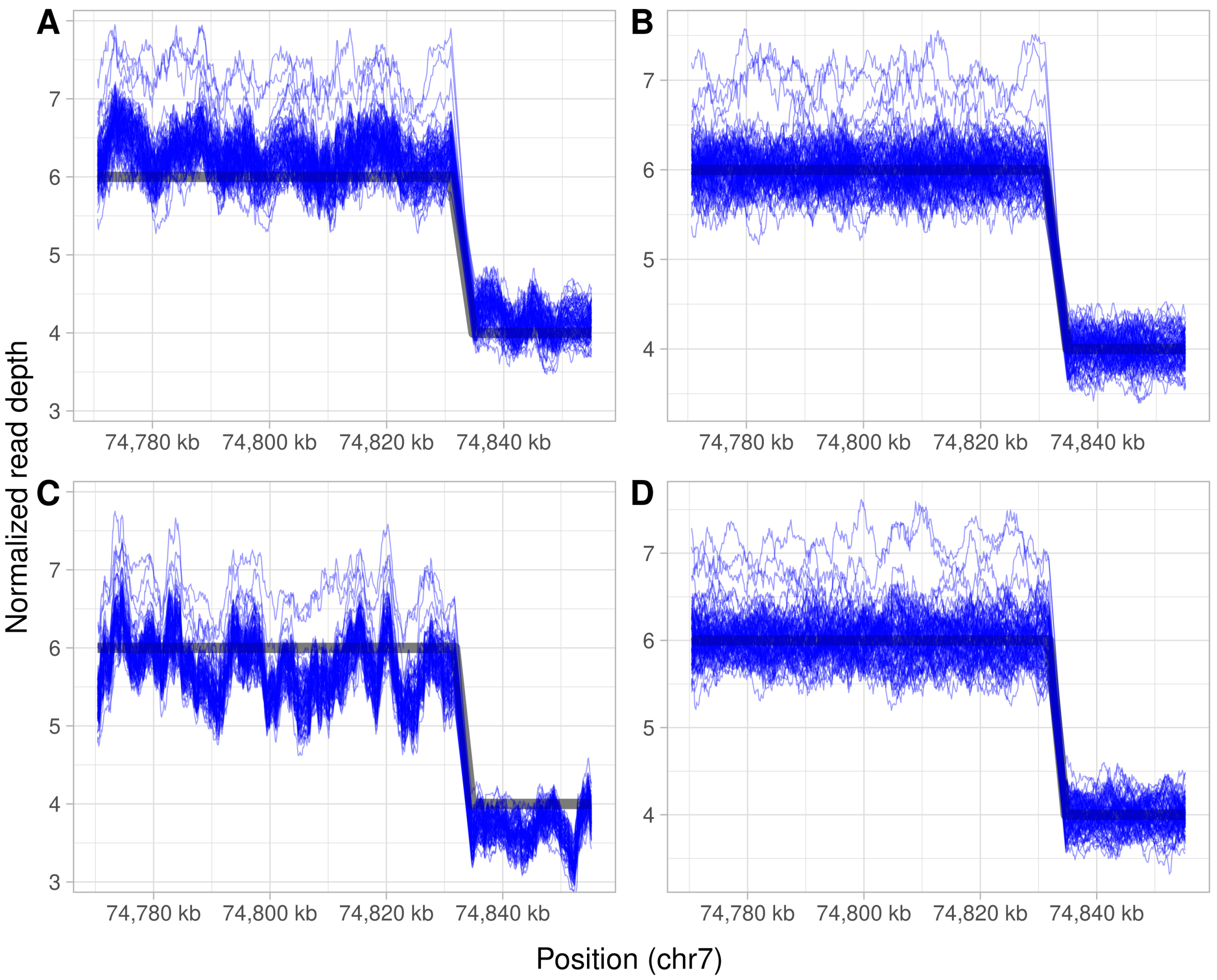

Supplementary Figure 5: **Normalized read depth in moving windows for 83 Han Chinese samples from IGSR and BGI datasets.** Each panel consists of 83 blue lines, each shows normalized read depth at three- and two-copy duplication chr7:74,769,000-74,856,500 that harbors NCF1 and GTF2IRD2 genes. Normalized read depth is averaged for each sample across moving 2,500 kb windows. Gray line shows reference copy number. (A) IGSR dataset: no read depth scaling. (B) IGSR dataset: scaling read depth based on window-specific multipliers m_w (same for all samples). (C) BGI dataset: no read depth scaling. (D) BGI dataset: scaling read depth based on window-specific multipliers m_w (same for all samples).

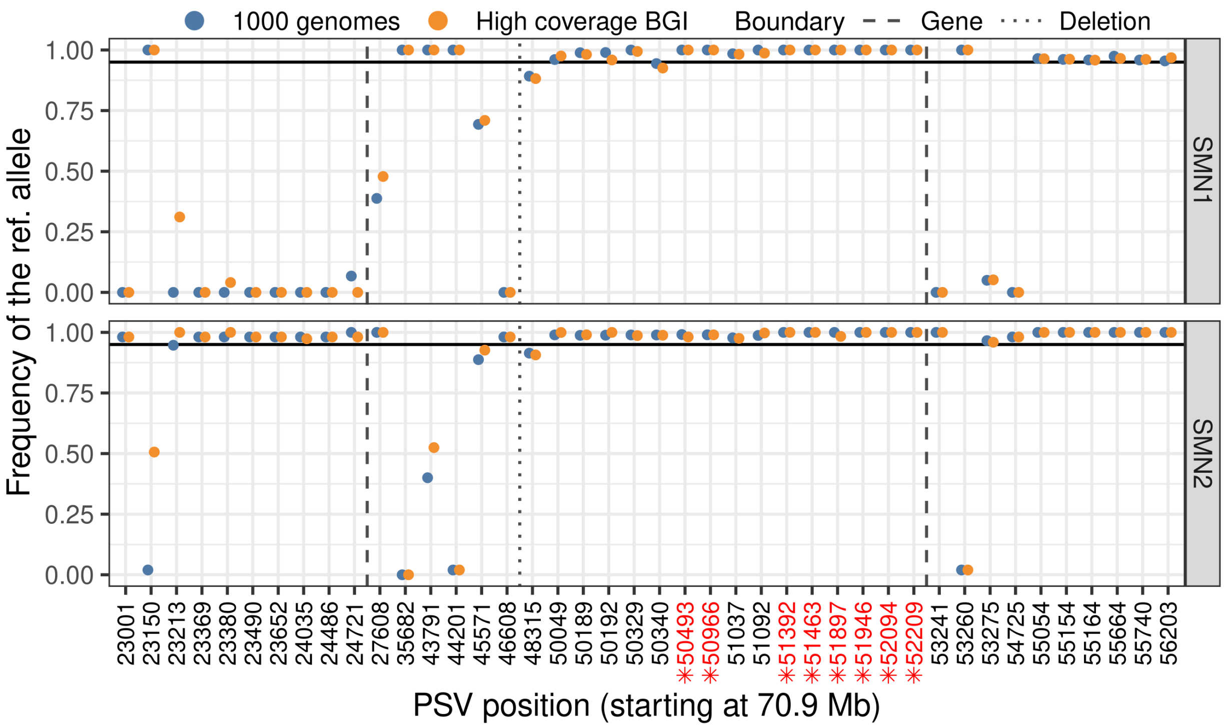

Supplementary Figure 6: **PSV f-values for the SMN1/2 locus estimated using Parascopy for the same set of 83 Han Chinese samples with two different WGS datasets.** The plot shows 42 PSVs in the vicinity of the SMN1 gene, of them 22 lie within SMN1. Horizontal black line shows reliability threshold (0.95), reliable PSVs have f-values over the threshold on both copies. Reliable PSVs used in SMNCopyNumberCaller are shown in red and marked by an asterisk.

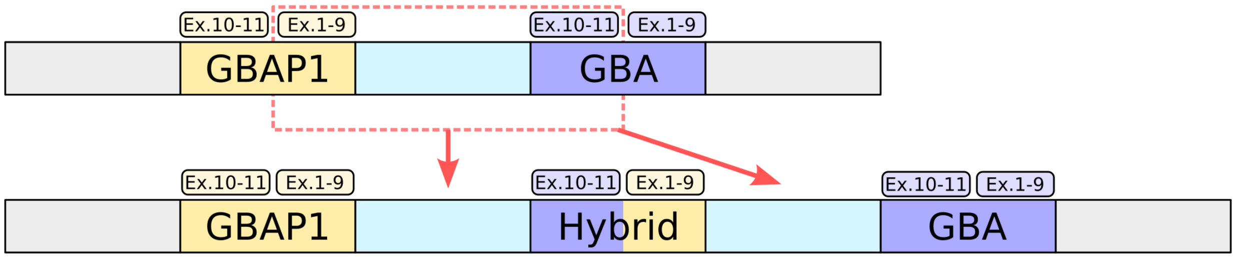

Supplementary Figure 7: **Structure of the duplication at the GBA locus.** The duplication affects a region between the GBAP1 pseudogene exons 1-9 and the GBA gene exons 10-11, including a region between GBAP1 and GBA, which is unique in the reference genome (shown in light blue). The duplication was constructed using a visual inspection of GBA locus *de-novo* assemblies. *De-novo* assemblies were obtained using SPAdes [5] based on the reads mapped to chr1:155,200,000-155,260,000 in two 1kGP samples (NA19031 and NA19159).

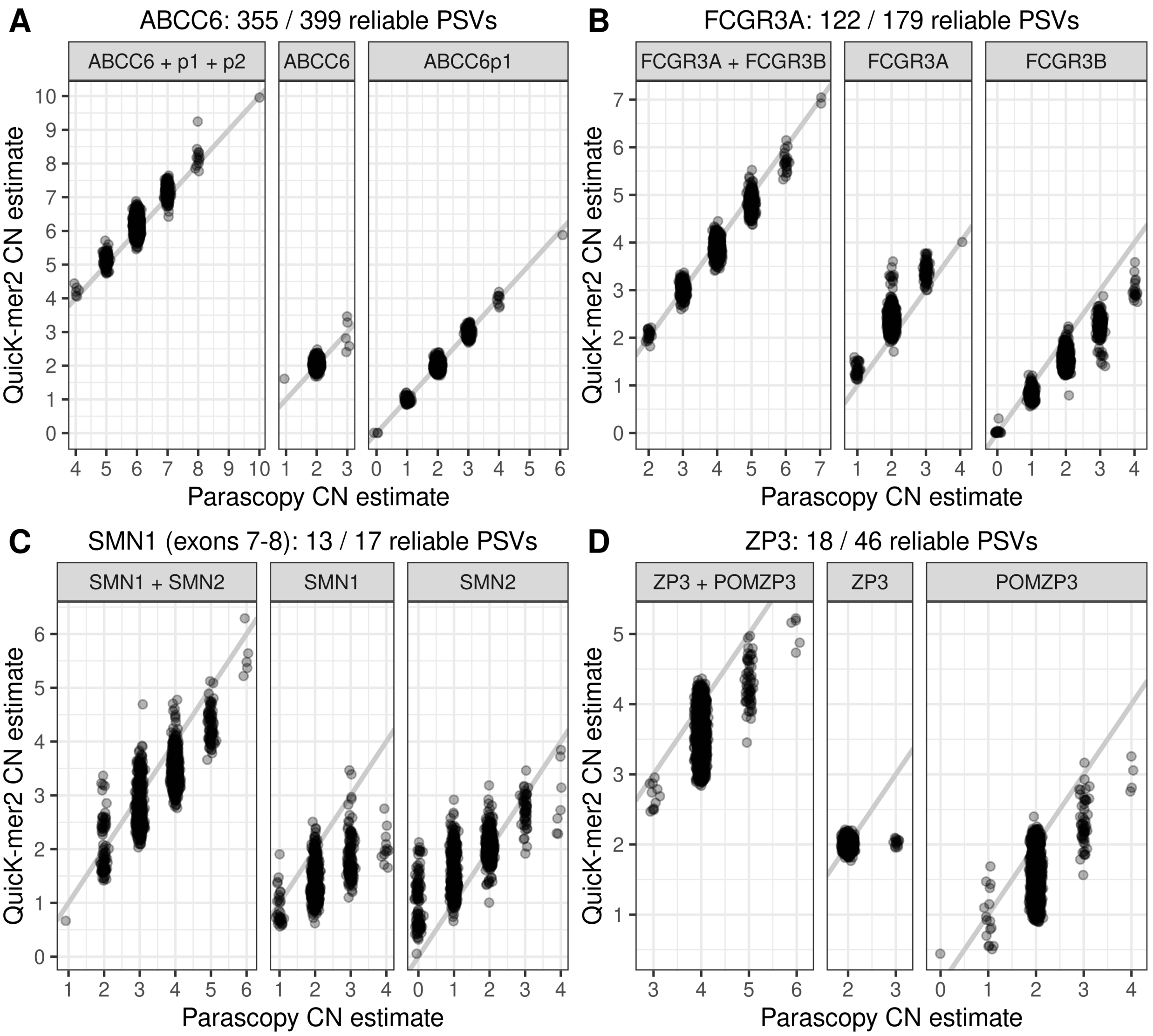

Supplementary Figure 8: **Comparison of Parascopy and QuicK-mer2 *AggregateCN* and *ParalogCN* estimates for four duplicated disease-associated genes: (A) ABCC6, (B) FCGR3A/B (B), (C) SMN1/2 and (D) ZP3/POMZP3.** The total number and number of reliable PSVs are also shown for each locus. For visual clarity, small jitter was added to the integer Parascopy copy number estimates.

Supplementary Table 1: **Accuracy of detecting partial deletions at the SMN1/2 and PMS2/PMS2CL loci using four different methods.** For the SMN1/2 partial deletion, 79/1109 samples were identified to carry the deletion event using MLPA data [6]. For the PMS2/PMS2CL deletion, 4/150 samples with deletions were identified using LR-PCR [2]. *AggregateCN* estimates at single positions within SMN1 exons 2 & 8 and PMS2 exons 14 & 15 were used for evaluation of each method.

Method	SMN1/2		PMS2/PMS2CL	
	Sensitivity	Specificity	Sensitivity	Specificity
Parascopy (Qual $\geq$ 20)	1.000	1.000	0.500	1.000
Parascopy (Qual $\geq$ 0)	1.000	1.000	1.000	1.000
SMNCopyNumberCaller	1.000	0.999	—	—
CNVnator (Qual $\geq$ 20)	0.797	0.850	0.500	0.743
CNVnator (Qual $\geq$ 0)	0.823	0.849	0.750	0.691
QuicK-mer2	0.709	0.382	0.000	1.000

Supplementary Table 2: **Concordance of Parascopy copy number estimates between replicate samples across 167 duplicated loci obtained using two independent sets of model parameters.**

Data type	Metric	(1) CHB	(2) IBS	(3) IBS 2/3	(4) IBS 1/3
AggregateCN	Available estimates (%)	96.3	95.9	94.0	88.3
	Concordance (%)	100.0	100.0	100.0	99.9
	Mean absolute difference	0.000	0.000	0.000	0.001
ParalogCN	Available estimates (%)	71.8	71.9	71.7	70.4
	Concordance (%)	98.9	98.9	98.8	98.4
	Mean absolute difference	0.043	0.033	0.034	0.038

(1) 103 Han Chinese samples analyzed using EAS and EUR model parameters
(2) 107 Iberian samples analyzed using EAS and EUR model parameters
(3-4) 107 Iberian samples subsampled to two-third & one-third coverage (EAS model parameters) and compared to full-coverage dataset (EUR model parameters)

2 Supplementary Methods

2.1 Creating homology table

The structure of the homology table is similar to the databases of segmental duplications that is available for human and other genome assemblies [7]. The motivation for constructing the homology table is to store extra information for pairs of homologous sequences and to additionally store short pairs of homologous sequences (< 1 kb) that are absent in segmental duplication databases.

To create the homology table, we split the reference genome into artificial reads of fixed length (900 bp) at a gap of every 150 bp and align the reads back to the reference genome using BWA [8]. For reads from repetitive regions, BWA reports the original location of the read as well as other regions in the genome, which we will call *homologous segments*. For each homologous sequence, we store the genomic coordinates, sequence alignment (CIGAR) and alignment strand. We filter homologous segments by length and sequence similarity (≥ 250 bp and $\geq 96\%$, by default). If the number of retained homologous segments for a region is more than a threshold (≥ 10 by default), we discard the read and mark its location as too complex for subsequent analysis.

Next, we combine overlapping read–homologous segment pairs into long *duplications*. To do that, we construct a directed graph where each node stores a read–segment pair (a read with n homologous segments would be represented by n nodes in the graph). We create an edge between nodes if both reads and their homologous segments overlap and are on the same strand. Direction of the edge is determined by the order of the reads and should match the order of the corresponding homologous segments.

We start the graph simplification by removing transitive edges. Next, we attempt to simplify the graph in cases where a node has two or more in- or out-edges. Without loss of generality, suppose there is a node with two or more out-edges and all homologous segments are on the "+" strand. The first node consists of the read $\tilde{a}$ and homologous segment $\tilde{b}$ and out-edges lead to nodes with reads $\{a_i\}$ and homologous segments $\{b_i\}$. We define two vectors of distances $d_{i1} = \mathrm{dist}(\tilde{a}, a_i)$ and $d_{i2} = \mathrm{dist}(\tilde{b}, b_i)$, where $\mathrm{dist}(x, y)$ represents the distance between starts of genomic regions x and y. In other words, d_{i1} and d_{i2} represent the jump between two consecutive reads and their homologous counterparts. We will also denote the length of the region x as $\mathrm{len}(x)$. Afterwards, we mark an edge i as *redundant* if at least one of the following statements is true:

- $d_{i1} > \min\{\mathrm{len}(\tilde{a}),\ \mathrm{len}(a_i)\} / 2$,
- $d_{i2} > \min\{\mathrm{len}(\tilde{b}),\ \mathrm{len}(b_i)\} / 2$,
- $\min\{d_{i1},\ d_{i2}\} > \max\{d_{i1},\ d_{i2}\} / 2$.

We then remove all redundant out-edges if at least one non-redundant out-edge remains. The reasoning behind this procedure is that if there are several possible continuations of the long homologous region, they must conflict with each other (because all transitive edges were already removed). This implies that there is a repeat, which is shorter than the artificial read size, and the reads or their homologous segments align to different copies of this repeat. In that case we want to keep an edge where jumps d_{i1} and d_{i2} are relatively small (first two statements), and similar in size (third statement). Note, that removed edges do not lead to removed nodes, so no information about additional small homologous regions is lost.

After graph simplification, we search for paths in the graph such that every node except the first has one in-edge, and every node except the last has one out-edge. We discard short paths (less than 4 nodes by default), if they do not form a separate connected component in the graph, and discard regions represented by these nodes from any further analysis. Finally, we combine a path of nodes into a single duplication. If an obtained duplication overlaps itself, we split it into several shorter duplications such that no duplication is self-overlapping.

2.2 Calculating background read depth

In order to accurately estimate copy number from read depth in repetitive regions of the genome, we calculate read depth in a large number of unique regions of the genome (reference copy number 2). In case of the human genome, we use a predetermined set of fixed-length (default = 100 bp) non-overlapping windows (≈ 90,000 windows for both hg19 and hg38 versions of the human genome). For other genomes, it is feasible to select a random set of non-overlapping fixed length windows outside repetitive regions.

For each window, the read depth is calculated by counting the number of read pairs for which the center of the first read lies within the window. This ensures that each read is counted once, and read counts from nearby windows are independent. For the same reason, we consider each paired-end read as a single entity. A window may have an abnormal read depth if it contains an insertion or deletion, overlaps a transposable element or other short duplication, or if it has low-complexity sequence. Such windows can skew the background read depth distribution. Therefore, we discard a window and call it *irregular* if at least 10% of reads in the window correspond to one of the three categories: reads with low mapping quality (< 10); reads not mapped in a proper pair; or reads with soft clipping at the ends. In addition, we also remove one adjacent window to the left and right of each *irregular* window.

For each sample and each GC-content value we aim to find a separate set of distribution parameters that would explain read depth in unique regions of the genome. To a select read depth distribution we evaluated Gaussian, Poisson and Negative Binomial distributions (Supp. Figure 4C-D) and found that Negative Binomial fits the observed values better, which is consistent with the previous studies [9, 10].

Next, we use LOWESS smoothing procedure [3, 4] to approximate read depth mean and variance for various GC-content values. As input, LOWESS takes a list of points (x, y) with the corresponding weights, if needed. As output, LOWESS provides a smoothed mean $\tilde{y}$ value for each requested $\tilde{x}$ value. Additionally, LOWESS has two parameters: local polynomial degree (we use degree 1) and a *fraction* parameter, which specifies that for each $\tilde{x}$ value only the closest *fraction* of the input points will be used. We run LOWESS smoothing procedure with $\tilde{x} = [0, 100] \cap \mathbb{Z}$ and the following parameters:

- **Read depth mean:** As input to LOWESS we use a set of points with x = window GC-content and y = window read depth (one point for each window). By default, we use *fraction* parameter = 0.1.
- **Read depth variance:** For read depth variance we cannot use individual read depth observations, therefore we create one point for each GC-content value, for which there are at least 10 windows. As y values we use read depth variance for windows with the corresponding GC-content, and we provide weight of the point based on the number of the corresponding windows. Here, we use *fraction* parameter = $2/3$.

As small number of genomic windows can lead to incorrect estimation of read depth mean and variance, we discard a set of very small and very large GC-content values and do not use windows with such GC values in copy number estimation. By default, we keep GC-content values u, for which there are both ≥ 1000 input genomic windows with GC-content values $\leq u$ and ≥ 1000 windows with values $\geq u$. This way, for the default set of 90,000 windows for the hg38 reference genome we keep GC-content values from 22 to 72 (Supp. Figure 4A-B).

For each non-discarded GC-content value we calculate Negative Binomial parameters as $n = \mu^2/(v - \mu)$ and $p = \mu/v$ according to the methods of moments [11], where μ and v are the read depth mean and variance approximations obtained using the LOWESS procedure. As Negative Binomial random variables must have variance greater or equal to the mean, we update variance as $v \leftarrow \max\{v, \mu + 0.001\}$ before calculating parameters n and p.

2.3 Re-mapping reads

In order to accurately calculate aggregate read depth and PSV-allelic read depth, we use a set of pooled reads. For each region R, we re-map reads from regions, homologous to R, back to R. We do that by utilizing sequence alignments between duplication copies, stored in the homology table. If a read is mapped to one of the duplication copies without gaps, read alignment to a different copy can be easily inferred using the sequence alignment between duplication copies (stored in the homology table). Otherwise, we merge the read alignment with the duplication sequence alignment in order to obtain a set of read positions matching second copy positions. Next, we fill the gaps in the alignment using Needleman-Wunsch algorithm [12]. Additionally, if the original read alignment contained soft clipping, we perform semi-global alignment [13] to check if the ends of the read can be aligned to the second copy. This procedure ensures that the vast majority of reads can be re-mapped without performing a full realignment of the read to the region R.

2.4 Finding aggregate copy number profiles

For a region group with reference copy number c_r we calculate aggregate copy number profiles using a matrix of aggregate read depth observations $\{o_w^{(s)}\}$ for all windows $w \in W$ and samples $s \in S$.

2.4.1 Estimating number of *AggregateCN* states

Average normalized aggregate read depth for a sample is calculated as

$$\overline{o}^{(s)} = \frac{1}{|W|} \sum_{w \in W} \frac{o_w^{(s)} \cdot 2 \cdot p_w^{(s)}}{n_w^{(s)} \cdot \left(1 - p_w^{(s)}\right)},$$

where $p_w^{(s)}$ and $n_w^{(s)}$ are Negative Binomial (NB) parameters for sample s and window w. Next, we select the minimum and maximum *AggregateCN* values:

$$c_1 = \max\left\{0,\ c_r - 2 \cdot B_l,\ \min\left\{c_r - B_l,\ \min_s \left\lfloor \overline{o}^{(s)} \right\rfloor - 1\right\}\right\},$$

$$c_K = \min\left\{c_r + 2 \cdot B_r,\ \max\left\{c_r + B_r,\ \max_s \left\lceil \overline{o}^{(s)} \right\rceil + 1\right\}\right\},$$

where B_l and B_r are bounds on how much copy number can differ from the reference copy number c_r, and are equal 5 and 7 by default, respectively. Range of *AggregateCN* values from c_1 to c_K will be used as a set of hidden states in *AggregateCN* Hidden Markov Model.

2.4.2 HMM definition

We define a homogeneous discrete-time HMM [14] for generating the read depth in T windows across a region with reference copy number c_r as follows:

1. For all windows in the region, we define a set of hidden states C, $|C| = K$ corresponding to K possible *AggregateCN* states. We denote hidden state of a sample s at window w as $Z_w^{(s)}$.
2. The initial state distribution $\pi_c = \max\left\{\sqrt{t}, \frac{1}{|S|}\right\}$ for *AggregateCN* $c \neq c_r$ and $\pi_{c_r} = 1 - \sum_{c \neq c_r} \pi_c$. Here and later we use an input parameter t, which is equal to 10^{-5} by default.

3. Transition parameters $\tilde{a}_{\nearrow w}$ and $\tilde{a}_{\searrow w}$ define all possible transitions (for fixed i and w):

$$a_{ijw} = \begin{cases} \tilde{a}_{\nearrow w}^{j-i} & \text{if } j > i, \\ \tilde{a}_{\searrow w}^{i-j} & \text{if } j < i, \\ 1 - \sum_{j \neq i} a_{ijw} & \text{if } j = i. \end{cases}$$

   On the first iteration $\tilde{a}_{\nearrow w} = \tilde{a}_{\searrow w} = t$ for all windows w. By default, we limit the maximal *AggregateCN* jump between two consecutive windows and set $a_{ijw} = 0$ if $|i - j| > 6$.

4. The emission probabilities are defined for each sample s using Negative Binomial parameters $n_w^{(s)}$ and $p_w^{(s)}$ corresponding to the sample s and GC-content of the window w. Using the fact that sum of NB-distributed random variables with parameters $(n_1,\ p)$ and $(n_2,\ p)$ is a NB random variable with parameters $(n_1 + n_2,\ p)$, we can multiply parameter n by the copy number in order to calculate emission probability of a certain hidden state. Therefore, we calculate emission probability of copy number c at window w as

$$b_w^{(s)}(c) = P_{\text{NB}} \left(o_w^{(s)};\ m_w \cdot n_w^{(s)} \cdot c/2,\ p_w^{(s)} \right)^{\psi(m_w)}$$

   where $o_w^{(s)}$ is the observed aggregate read depth. We divide c by 2 as background read depth was calculated for regions with copy number 2. For copy number zero we use $c = 0.01$ to allow possible errorneous read alignments. m_w is a scale parameter and $\psi\,(m_w)$ is the scale parameter weight, both are equal to one on the first iteration.

2.4.3 Updating emission probabilities using scale parameters

After the first iteration we introduce non-trivial multipliers m_w for each window w. This scale parameter is used to remove window-specific sequencing bias that is shared across all samples. Windows with large bias should contribute less to the likelihood, so we assign a weight to the scale parameter based on the distance $|m_w - 1|$ ($m_w = 1$ represents no significant bias). For a region with reference copy number c_r we expect that all scale parameters should be within $(\frac{c_r - 1}{c_r},\ \frac{c_r + 1}{c_r})$, so scale parameters outside these bounds are assigned weight 0. For scale parameters within the bounds we distribute weights according to the tricube kernel [15]:

$$\psi(m_w) = \left(1 - \min(1, |m_w - 1| \cdot c_r)^3\right)^3.$$

On each HMM iteration we run the Forward-Backward algorithm [16] to obtain a range of matrices $\gamma_{c,w}^{(s)} = P\left(Z_w^{(s)} = c \,\middle|\, o_{1:T}^{(s)}\right)$ — probability of sample s having copy number c at window w. We use γ to update the scale parameters:

$$m_w \leftarrow \underset{m}{\operatorname{argmax}} \prod_{s \in S} \sum_{c \in C} \gamma_{c,w}^{(s)} \cdot P_{\text{NB}} \left(o_w^{(s)};\ m_w \cdot n_w^{(s)} \cdot c/2,\ p_w^{(s)} \right),$$

where S is the set of all samples and C is the set of hidden states (and copy number values they represent).

2.4.4 Updating initial and transition probabilities

In addition to probabilities matrices γ, the Forward-Backward algorithm provides matrices $\alpha_{c,w}^{(s)} = P\left(o_{1:w}^{(s)},\ Z_w^{(s)} = c\right)$ and $\beta_{c,w}^{(s)} = P\left(o_{w+1:T}^{(s)} \,\middle|\, Z_w^{(s)} = c\right)$ — forward and backward probabilities,

respectively. Then, total probability of a sample s is $\tau(s) = P\left(o^{(s)}_{1:T}\right) = \sum_{c\in C} \alpha^{(s)}_{c,T}$. According to the Baum-Welch algorithm [14], we update initial probabilities as

$$\pi_c \leftarrow \frac{1}{|S|} \sum_{s\in S} \gamma^{(s)}_{c,1}.$$

Additionally, we bound $\pi_c \leftarrow \max\left\{\pi_c, \sqrt{t}, \frac{1}{|S|}\right\}$.

Let $\xi^{(s)}_{w,i,j}$ denote the probability of going from state i at window w to state j at window $w+1$ at the sample s. It can be calculated as

$$\xi^{(s)}_{ijw} = \frac{1}{\tau(s)} \cdot \alpha^{(s)}_{i,w} \cdot a_{ijw} \cdot \beta^{(s)}_{j,w+1} \cdot b^{(s)}_{w+1}(j),$$

where a_{ijw} denotes transition probability between hidden states i and j at windows w and $w+1$. Similarly, we can calculate the probability of increasing or decreasing copy number:

$$\xi^{(s)}_{\nearrow w} = \sum_{i\in C,\ j\in C,\ j>i} \xi^{(s)}_{ijw},$$

$$\xi^{(s)}_{\searrow w} = \sum_{i\in C,\ j\in C,\ j<i} \xi^{(s)}_{ijw}.$$

Next, we average these values across all samples to get probabilities of increasing or decreasing aggregate copy number:

$$\tilde{a}_{\nearrow w} = \frac{1}{|S|} \sum_{s\in S} \xi^{(s)}_{\nearrow w}, \qquad \tilde{a}_{\searrow w} = \frac{1}{|S|} \sum_{s\in S} \xi^{(s)}_{\searrow w}.$$

2.4.5 Speeding up HMM convergence

In some cases, the iterative HMM converges slowly. Therefore, to speed up the conversion we search for peaks in $\tilde{a}_{\nearrow w}$ and $\tilde{a}_{\searrow w}$. We define peaks as local maxima higher than t and higher than any other values in the 10 window neighbourhood to the left and right. For each peak we set $\tilde{a}$ to the sum of $\tilde{a}$ over the neighbourhood of the peak, and decrease $\tilde{a}$ values in the neighbourhood to t. Next, we bound $\tilde{a}_{\nearrow w}$ and $\tilde{a}_{\searrow w}$ to be at least t and at most 0.1, and set new transition probabilities a_{ijw} based on $\tilde{a}_{\nearrow w}$ and $\tilde{a}_{\searrow w}$ as described above.

2.4.6 Log-likelihood convergence

Similar to most HMM applications, we aim to repeat HMM parameter refinement until the log-likelihood $\mathscr{L} = \sum_{s\in S} \log \tau(s)$ stops increasing. However, the emission probabilities definition includes the scale parameter weight exponent $\psi(m_w)$, which equals to one for all windows on the first iteration and can be lower on the subsequent iterations. This can lead to a drop in log-likelihood after the first iteration. Therefore, we allow the log-likelihood to decrease between the first and second iteration, and stop only after third iteration if the increase in the log-likelihood is less than 0.01.

2.4.7 Aggregate copy number quality

To assign a quality to each *AggregateCN* estimate, we calculate a probability of the prediction and probabilities of alternative predictions. Suppose, the Viterbi path [17] for a sample s contains a

constant stretch between windows u and v with $Z^{(s)}_{u:v} = c$. We calculate the probability of such a stretch using forward and backward probability matrices $\alpha^{(s)}$ and $\beta^{(s)}$:

$$P\left(Z^{(s)}_{u:v} = c \,\middle|\, o^{(s)}_{1:T}\right) = \frac{1}{\tau(s)} \cdot \alpha^{(s)}_{c,u} \cdot \beta^{(s)}_{c,v} \cdot \prod_{k=u}^{v-1} a_{cck} \cdot b_{k+1}(c).$$

Next, we calculate probabilities of the alternative non-overlapping paths to calculate the probability of an error:

$$P_{\text{error}} = 1 - \frac{P\left(Z^{(s)}_{u:v} = c \,\middle|\, o^{(s)}_{1:T}\right)}{\sum_{c' \in C} P\left(Z^{(s)}_{u:v} = c' \,\middle|\, o^{(s)}_{1:T}\right)}.$$

Finally, *AggregateCN* prediction quality is calculated as a Phred [18] quality score: $-10 \cdot \log_{10} P_{\text{error}}$.

2.5 Estimating paralog-specific copy number using PSVs

For a sample s and PSV v we observe allele counts X_{sv}, which form the observed data X. Note that X_{sv} is a tuple as it stores read counts for two or more alleles of the PSV. We can easily calculate probability $P(X_{sv} \mid \widehat{G}_v = \hat{g})$ of allele counts given the PSV genotype $\hat{g}$ using multinomial distribution [19,20].

In a region with reference copy number c_r, each PSV v is assigned a vector $f_v \in [0,1]^{c_r/2}$, where f_{vk} represents the frequency of the reference allele on k-th copy of the duplication across the whole population (there are total $c_r/2$ copies). For example, a PSV has reference allele A on the first copy and C on the second copy, but all reads in all samples support C on both copies. In that case $f_{v,1} = 0$ and $f_{v,2} = 1$ as the frequency of the reference allele is 0 on the first copy.

To calculate the probability of observing read counts X_{sv} in case of the sample paralog-specific copy number *(ParalogCN)* g:

$$P(X_{sv} \mid G_s = g, f_v) = \sum_{\hat{g}} P(X_{sv} \mid \widehat{G}_v = \hat{g}) \cdot P(\widehat{G}_v = \hat{g} \mid G_s = g, f_v).$$

and to calculate probability $P(\widehat{G}_v = \hat{g} \mid G_s = g, f_v)$ of observing PSV genotype $\hat{g}$ given the *ParalogCN* g and frequencies f_v we need to calculate coefficients in a multivariate polynomial. For example, in a 2-copy duplication ($c_r = 4$) PSV v has two values $f_1 = f_{v,1}$ and $f_2 = f_{v,2}$. Then, if sample paralog-specific copy number $g = 2{,}2$ (each copy is represented twice) we need to expand a polynomial

$$\left(f_1 \cdot u_1 + (1 - f_1) \cdot u_2\right)^2 \cdot \left((1 - f_2) \cdot u_1 + f_2 \cdot u_2\right)^2.$$

Then the probability $P(\widehat{G}_v = \hat{g} \mid G_s = g, f_v)$ of PSV genotype $\hat{g}$ is a coefficient in front of $u_1^{\hat{g}_1} \cdot u_2^{\hat{g}_2}$. For example, probability of a PSV genotype $\hat{g} = 4{,}0$ would be $f_1^2 \cdot (1 - f_2)^2$. This can be generalized for any PSV genotype, any paralog-specific, aggregate and reference copy numbers. Additionally, we use f as variables (instead of numeric values) to expand the multivariate polynomial (with variables $u_{\ldots}$ and $f_{\ldots}$) in advance, which significantly speeds up PSV genotype probability calculation.

2.5.1 EM algorithm

Next, we define the total likelihood of the model $L(X, f) = \prod_{s \in S} P(X_s \mid f)$ as the product of probabilities for all samples. Probability of a single sample is $P(X_s \mid f) = \sum_g P(X_s, G_s = g \mid f)$, and probability of a sample and a *ParalogCN* g is

$$P(X_s, G_s = g \mid f) = p(g) \prod_{v \in V} P(X_{sv} \mid G_s = g, f_v),$$

where V is a total set of PSVs and $p(g)$ is a paralog-specific copy number prior. We define *ParalogCN* priors based on the distance to the reference *ParalogCN* (all copies are represented twice), with the smallest prior = 10^{-6} assigned to the most extreme *ParalogCN*s. For example, for a two-copy duplication *ParalogCN* 4,0 is assigned a prior 10^{-6}, *ParalogCN* 3,1 is assigned a prior 10^{-3} and *ParalogCN* 2,2 is set to 1 minus all other priors.

According to the Expectation-Maximization algorithm [21], on the **E-step** we find the distribution of the hidden variables (paralog-specific copy numbers):

$$P(G_s = g \mid X_s, f) = \frac{P(X_s, G_s = g \mid f)}{\sum_{g'} P(X_s, G_s = g' \mid f)}.$$

We will denote $P(G_s = g \mid X_s, f)$ as $\zeta_{s,g}$.

Next, during the **M-step** we maximize log-likelihood:

$$f^\star \leftarrow \operatorname*{argmax}_f \; p(f) \cdot \sum_{s \in S} \zeta_{s,g} \cdot \log \frac{P(G_s = g \mid X_s, f)}{\zeta_{s,g}}.$$

This step can be solved numerically and independently for all PSVs. We select prior $p(f)$ in a way to encourage higher values of f:

$$p(f) = \prod_{v \in V} P_{\text{Beta}} \left(x \in \left[\max_k(f_{vk}) - 10^{-6},\ \max_k(f_{vk}) \right];\ \alpha = 5,\ \beta = 1 \right).$$

After the EM algorithm converges, we use frequency matrix f to select a set of reliable PSVs $\left\{ v \,\middle|\, \min_{k=1}^{c_r/2} f_{vk} \geq 0.95 \right\}$. We use reliable PSVs to calculate probability of all *ParalogCN* values for all samples, including samples with non-reference *AggregateCN*. Next, for each duplication copy we calculate marginal probability over all *ParalogCN* probabilities:

$$P([G_s]_k = c \mid X_s, f) = \sum_{g \text{ s.t. } g_k = c} P(G_s = g \mid X_s, f).$$

This allows us to calculate most likely *ParalogCN* value $c = \operatorname{argmax}_{c'} P([G_s]_k = c' \mid X_s, f)$ for each duplication copy, and to calculate the corresponding Phred [18] quality score $-10 \cdot \log_{10} P([G_s]_k \neq c \mid X_s, f)$. Additionally, we do not assign copy number to some duplication copies if the quality is near zero (< 5). For example a sample in three-copy duplication can have a paralog-specific copy number (2,?,?), when we do not have enough information to distinguish between the second and the third copies.

2.5.2 Information content of the PSVs

In certain cases, the EM algorithm may converge to an undesirable solution. For example, suppose that there is a set of unreliable PSVs V_{unrel} that exhibit PSV genotype 4,0 in all samples (all reads support an allele corresponding to the first copy of the duplication). Additionally, there is a smaller set of reliable PSVs V_{rel} that exhibit PSV genotype 2,2 (alleles from both copies are present with equal proportions). This situation occurs in the two-copy duplication that includes genes *SERF1A* and *SMN1*. Depending on the relative sizes of PSV sets V_{unrel} and V_{rel}, the EM algorithm can converge to two possible solutions:

- Assign $f_{v,1} \simeq 1$ for all $v \in V_{\text{unrel}}$ and predict *ParalogCN* $= 4, 0$ for all samples. In that case the EM algorithm would assign $f_{v',1} \simeq 1/2$ for all $v' \in V_{\text{rel}}$. Note that $f_{v,2}$ can be anything for both PSV sets, as there are no samples that have a second copy. This solution can be explained in the following way: for every sample there are four haplotypes, all of which are more similar to the first copy of the duplication than to the second, therefore it would be correct to set *ParalogCN* $= 4, 0$ for all samples.

- Assign $f_{v,2} = 0$ for all $v \in V_{\text{unrel}}$ (frequency of the reference allele on the second copy is 0). In this solution $f_{v,1} \simeq 1$ for $v \in V_{\text{unrel}}$ and $f_{v',1} \simeq f_{v',2} \simeq 1$ for $v' \in V_{\text{rel}}$; *ParalogCN* prediction for all samples would be 2,2. This solution is more appropriate in the following way: even though four haplotypes of each sample are more similar to the first copy, two of them are significantly different from the other two (at reliable PSVs V_{rel}), and share many similarities with the second copy of the duplication. Therefore this solution provides more information.

To encourage the EM algorithm to converge to the second solution we add a weight to each PSV based on multiple samples and call it *information content* of the PSV. Suppose a PSV v has n alleles, then

$$I(v) = \frac{1}{|S|} \sum_{s \in S} \sum_{\hat{g}} P(\widehat{G}_v = \hat{g} \mid X_{sv}) \cdot \frac{\sum_{k=1}^{n} \mathbb{1}\left[\hat{g}_k \neq 0\right] - 1}{n - 1}$$

The fraction on the right represents how well PSV alleles are represented in the PSV genotype. For example, PSV genotypes of a PSV with three alleles would have the following weights: 1 for genotypes without zeros, such as (2,2,2), (3,2,1); 1/2 for genotypes with one zero, such as (4,2,0), (3,3,0); 0 for genotypes with two zeros, such as (6,0,0), (0,6,0) and (0,0,6). This way, if some allele of a PSV is consistently missing in many samples — the PSV would get low information content I.

During the E-step (calculating sample *ParalogCN* probabilities) we use the PSV information content as an exponent:

$$P(X_s, G_s = g \mid f) = p(g) \prod_{v \in V} P^{\,I(v)}(X_{sv} \mid G_s = g, f_v),$$

This forces PSVs with information content close to 0 have a very small effect on the paralog-specific copy number calculation and on the total likelihood.

2.5.3 Selecting starting states for EM algorithm

A single read can cover several PSVs if they are close enough. Therefore, we filter out PSVs if they are closer than 100 bp to each other. While discarding neighboring PSVs we first remove PSVs with low information content, and then remove PSVs that represent insertions or deletions.

The EM algorithm is not guaranteed to reach the global maximum and can be trapped at local maxima. A standard approach to avoid local maxima is to use several starting solutions. We cluster the PSVs to obtain several starting positions for the EM algorithm as follows: for each PSV v we have a vector of allele counts $\{X_{sv}\}_{s \in S}$, which we then transform into a numeric vector by calculating the fraction of the allele corresponding to the first copy: $\{[X_{sv}]_1 / \sum X_{sv}\}_{s \in S}$. Next, we construct a Pearson correlation matrix over PSVs and split it into two clusters based on the hierarchical clustering [22]. Then we use three starting PSV sets: the two separate clusters and all PSVs together. We start by assigning $f_{v,\cdot} = 0.9$ to PSVs in the starting set and $f_{v,\cdot} = 0.5$ to all other PSVs. Next, we iteratively run E- and M-steps until the algorithm converges and finally select the result from the starting set that produced the highest total likelihood.

2.6 Extending homology table to include an additional repeat copy for *OTOA*

Analysis of the two-copy *OTOA* locus on chromosome 16 using Parascopy showed that majority of the samples have three duplication copies. More than 75% and 91% samples in the European and African continental populations had *AggregateCN* = 6. Alignment of the *OTOA* and *OTOAP1* (pseudo-gene) sequences to the recently generated high-quality genome assembly for a human cell line, CHM13 [23] using Minimap2 [24] generated three independent hits for both sequences. We denote the hit that is least similar to *OTOA* and *OTOAP1* as *OTOAP** and add it to the homology table. It is not required to create the whole homology table anew to do this — we find sequence homologies between the *OTOAP** sequence and hg38 reference genome and add them to the

homology table. This allows us to find reliable PSVs that distinguish *OTOA* and *OTOAP** and detect paralog-specific copy number of an extended three-copy duplication.

Next, to analyze the new three-copy duplication, we can use the standard aligment files mapped to the hg38 reference genome, which contain reads mapped to *OTOA* and *OTOAP1*, but not to *OTOAP**. Since Parascopy first re-maps reads from different repeat copies to a single copy, it is not important to have correct read alignments to all three copies.

2.7 Subsampling reads

In order to estimate the impact of read depth on the accuracy of Parascopy, we artificially reduced coverage for 107 samples from the Iberian population (IBS) from the 1kGP WGS data. Since the 1kGP samples were sequenced to an average read depth of 33×, we randomly and independently selected read pairs with probabilities 1/3 and 2/3 to create subsampled datasets with average read depth 11× and 22×.

2.8 Paralog-specific copy number validation using trios

To determine trio concordance for *ParalogCN* values, it is useful to model the *ParalogCN* for each homologous chromosome or haplotype. Suppose an individual has c copies of a repeat copy R ($ParalogCN_R = c$), then one of the homologous chromosome has $a \in [0, c]$ copies of R, while the other homologous chromosome would have $b = c - a$ copies of R. In a sample that is concordant with the reference, repeat copy R appears once on each homologous chromosome, i.e. $c = 2$ and $a = b = 1$. Here (a, b) is the *diploid ParalogCN* at the repeat copy R for the individual.

Suppose the diploid *ParalogCN* at the repeat copy R for two parents is (a_m, b_m) and (a_f, b_f). Then the four possible diploid *ParalogCN* values for a child are: (a_m, a_f), (a_m, b_f), (b_m, a_f) and (b_m, b_f). Note that this does not model situations when the different copies of R lie on several non-homologous chromosomes or lie far from each other on the same homogolous chromosome, in both cases a child can receive a different combination of copies of R.

In a sample set with n individuals, let n_c be the number of samples with c copies of R (summed over two homologous chromosomes) and let $c_{\max} = \operatorname{argmax}_c n_c$ be the maximal observed copy number. Suppose vector ϕ stores the allele frequency distribution of copy number values for a single homologous chromosome in the population. If the two homologous chromosomes are independent, the expected number of samples with $ParalogCN_R = c$ would be $e_c = \sum_{a=0}^{c} n \cdot \phi_a \cdot \phi_{c-a}$. We calculate the likelihood of the copy number frequencies ϕ using chi-square distribution with $c_{\max}$ degrees of freedom and

$$\chi^2 = \sum_{c=0}^{c_{\max}} \frac{(e_c - n_c)^2}{e_c}.$$

Next, we use maximum likelihood to estimate the most-likely frequencies ϕ according to the observed diploid $ParalogCN_R$ counts in each continental population. On all steps, only samples with high-quality (≥ 20) $ParalogCN_R$ estimates were used.

Throughout 5 continental populations, 167 duplicated loci and a total of 384 paralogs (1920 total entries), we estimated copy number frequencies in 1474 cases with at least 10 samples. In only 20 cases χ^2 p-value was under 0.05, and in only 4 cases (0.3%) p-values were under 0.05 after the Benjamini-Hochberg correction [25] (controlling false-discovery rate). This shows that (i) probabilistic model is consistent with *ParalogCN* estimates; (ii) Parascopy *ParalogCN* values do not violate Hardy–Weinberg equilibrium in the vast majority of the cases.

Using the distribution ϕ_c, we can calculate the probability of observing a child with c copies

of R when the two parents have c_m and c_f copies:

$$p = P(c \mid c_m, c_f) = \sum_{\substack{a_m+b_m=c_m \\ a_m, b_m \geq 0}} P\left(ParalogCN_R = (a_m,\ b_m)\right) \times \sum_{\substack{a_f+b_f=c_f \\ a_f, b_f \geq 0}} P\left(ParalogCN_R = (a_f,\ b_f)\right) \times \sum_{\substack{x \in \{a_m, b_m\} \\ y \in \{a_f, b_f\}}} \frac{1}{4} \cdot \mathbb{1}[x + y = c].$$

The probability $P\big(ParalogCN_R = (a,\ b)\big)$ of having diploid *ParalogCN* at the repeat copy R can be calculated as:

$$P\big(ParalogCN_R = (a,\ b)\big) = \frac{\phi_a \cdot \phi_b}{\sum_{a'+b'=a+b} \phi_{a'} \cdot \phi_{b'}}.$$

A low value of probability p implies that the child's *ParalogCN* estimate is not consistent with the parental *ParalogCN* values. We use a default threshold of $p < 0.01$ to identify discordant trios. Stricter thresholds (such as $p < 0.05$ and $p < 0.1$) produce similar results.

Supplementary References

[1] Shebanits, K. et al. Copy number determination of the gene for the human pancreatic polypeptide receptor NPY4R using read depth analysis and droplet digital PCR. *BMC Biotechnol.* **19**, 31 (2019).

[2] Gould, G. M. et al. Detecting clinically actionable variants in the 3′ exons of PMS2 via a reflex workflow based on equivalent hybrid capture of the gene and its pseudogene. *BMC Med. Genet.* **19**, 1–13 (2018).

[3] Cleveland, W. S. Robust locally weighted regression and smoothing scatterplots. *J. Am. Stat. Assoc.* **74**, 829–836 (1979).

[4] Cleveland, W. S. LOWESS: A program for smoothing scatterplots by robust locally weighted regression. *Am. Stat.* **35**, 54 (1981).

[5] Prjibelski, A., Antipov, D., Meleshko, D., Lapidus, A. & Korobeynikov, A. Using spades de novo assembler. *Curr. Protoc. Bioinform.* **70**, e102 (2020).

[6] Vijzelaar, R. et al. The frequency of SMN gene variants lacking exon 7 and 8 is highly population dependent. *PLoS One* **14**, e0220211 (2019).

[7] Karolchik, D. et al. The UCSC Table Browser data retrieval tool. *Nucleic Acids Res.* **32**, D493–D496 (2004).

[8] Li, H. & Durbin, R. Fast and accurate short read alignment with Burrows–Wheeler transform. *Bioinformatics* **25**, 1754–1760 (2009).

[9] Miller, C. A., Hampton, O., Coarfa, C. & Milosavljevic, A. ReadDepth: a parallel r package for detecting copy number alterations from short sequencing reads. *PloS One* **6**, e16327 (2011).

[10] Sampson, J., Jacobs, K., Yeager, M., Chanock, S. & Chatterjee, N. Efficient study design for next generation sequencing. *Genet. Epidemiol.* **35**, 269–277 (2011).

[11] Savani, V. & Zhigljavsky, A. A. Efficient estimation of parameters of the negative binomial distribution. *Commun. Stat. — Theory Methods* **35**, 767–783 (2006).

[12] Needleman, S. B. & Wunsch, C. D. A general method applicable to the search for similarities in the amino acid sequence of two proteins. *J. Mol. Biol.* **48**, 443–453 (1970).

[13] Smith, T. F., Waterman, M. S. et al. Identification of common molecular subsequences. *J. Mol. Biol.* **147**, 195–197 (1981).

[14] Baum, L. E. & Petrie, T. Statistical inference for probabilistic functions of finite state Markov chains. *Ann. Math. Stat.* **37**, 1554–1563 (1966).

[15] Altman, N. S. An introduction to kernel and nearest-neighbor nonparametric regression. *Am. Stat.* **46**, 175–185 (1992).

[16] Stratonovich, R. L. Conditional markov processes. *Non-linear Transformations of Stochastic Processes*, 427–453 (Elsevier, 1965).

[17] Viterbi, A. Error bounds for convolutional codes and an asymptotically optimum decoding algorithm. *IEEE T. on Information Theory* **13**, 260–269 (1967).

[18] Ewing, B. & Green, P. Base-calling of automated sequencer traces using phred. II. Error probabilities. *Genome Res.* **8**, 186–194 (1998).

[19] Goya, R. et al. SNVMix: predicting single nucleotide variants from next-generation sequencing of tumors. *Bioinformatics* **26**, 730–736 (2010).

[20] Hohenlohe, P. A. et al. Population genomics of parallel adaptation in threespine stickleback using sequenced RAD tags. *PLoS Genet.* **6**, e1000862 (2010).

[21] Dempster, A. P., Laird, N. M. & Rubin, D. B. Maximum likelihood from incomplete data via the EM algorithm. *J. R. Stat. Soc. Ser. B Methodol.* **39**, 1–22 (1977).

[22] Müllner, D. fastcluster: Fast hierarchical, agglomerative clustering routines for R and Python. *J. Stat. Softw.* **53**, 1–18 (2013).

[23] Nurk, S. et al. The complete sequence of a human genome. *Science* **376**, 44–53 (2022).

[24] Li, H. Minimap2: pairwise alignment for nucleotide sequences. *Bioinformatics* **34**, 3094–3100 (2018).

[25] Benjamini, Y. & Hochberg, Y. Controlling the false discovery rate: a practical and powerful approach to multiple testing. *J. R. Stat. Soc. Ser. B Methodol.* **57**, 289–300 (1995).
