# Supplementary material for: Robust and accurate estimation of paralog-specific copy number for duplicated genes using whole-genome sequencing
Source: Nat Commun. 2022 Jun 9;13:3221. doi: 10.1038/s41467-022-30930-3 (PMC9184528; doi:10.1038/s41467-022-30930-3)
Supplement: Supplementary file 3 — Description of Additional Supplementary Files [file 41467_2022_30930_MOESM3_ESM.pdf]

Description of Additional Supplementary Files

Name: Supplementary Data 1
Description: PSV reliability in the SMN1/2 duplication across 5 continental populations. The table contains 87 PSVs in 2-copy duplication that contains SMN1/2 genes. Two values are associated with each PSV and each population: f-values on SMN1 and SMN2 copies of the duplication.

Name: Supplementary Data 2
Description: List of experimental copy number datasets and loci used for validation.

Name: Supplementary Data 3
Description: 167 low-copy repeat loci used for Parascopy evaluation. For each locus the table contains its reference copy number, genes that overlap the locus and genes that overlap regions, homologous to the locus. If a locus contains subregions with various reference copy numbers, they are displayed through a comma (subregions with reference copy number = 2 are ignored).

Name: Supplementary Data 4
Description: Percentage of concordant trios across 137 duplicated loci. For each duplicated locus and each paralog the table contains the total number of samples with highquality ParalogCN values, ParalogCN mean and standard deviation. For each continental population, it contains number of trios with high-quality ParalogCN estimates and number of trios with concordant ParalogCN estimates. Additionally, the table shows "chi-square goodness of Fit p-values" for population copy number frequencies. 30/167 input loci have no trios with high-quality ParalogCN estimates, and therefore contain no information about concordant trios (reported as NA).

Name: Supplementary Data 5
Description: Number of samples with high-quality AggregateCN and ParalogCN estimates for each locus. The table shows analysis of the copy number estimates across 2504 samples from 5 continental populations for 167 loci. Copy number estimates were selected based on a single point for each locus. AggregateCN estimates are said to be high quality if they pass a quality threshold of 20. ParalogCN estimates are said to be high quality if AggregateCN estimate is high quality, ParalogCN filter is PASS and ParalogCN quality is at least 20 at each copy.

Name: Supplementary Data 6
Description: PSV reliability across 326 duplicated genes. The table reports the percentage of reliable PSVs for each continental population and each gene that has a PSV within 167 input loci. Of of 326 duplicated genes in the table, 214 genes are protein coding and 84 are disease-associated.

Name: Supplementary Data 7
Description: List of single positions within duplicated loci used for Parascopy validation. As output, Parascopy provides an AggregateCN/ParalogCN profile for each sample and each locus, which may consist of multiple disjoint subregions with different copy number

estimates. For comparison with experimental copy number, we used copy number observations that overlap a list of positions in this table, which leads to no more than one AggregateCN and ParalogCN observation for each sample and locus. Each position in the list lies within a duplicated exon of the corresponding gene.
