# Supplementary material for: Robust and accurate estimation of paralog-specific copy number for duplicated genes using whole-genome sequencing
Source: Nat Commun. 2022 Jun 9;13:3221. doi: 10.1038/s41467-022-30930-3 (PMC9184528; doi:10.1038/s41467-022-30930-3)
Supplement: Supplementary file 5 — Reporting Summary [file 41467_2022_30930_MOESM5_ESM.pdf]

nature portfolio

Corresponding author(s): Vikas Bansal
Last updated by author(s): May 12, 2022

Reporting Summary

Nature Portfolio wishes to improve the reproducibility of the work that we publish. This form provides structure for consistency and transparency in reporting. For further information on Nature Portfolio policies, see our Editorial Policies and the Editorial Policy Checklist.

Statistics

For all statistical analyses, confirm that the following items are present in the figure legend, table legend, main text, or Methods section.

n/a	Confirmed	
☐	☒	The exact sample size (*n*) for each experimental group/condition, given as a discrete number and unit of measurement
☒	☐	A statement on whether measurements were taken from distinct samples or whether the same sample was measured repeatedly
☐	☒	The statistical test(s) used AND whether they are one- or two-sided<br>*Only common tests should be described solely by name; describe more complex techniques in the Methods section.*
☒	☐	A description of all covariates tested
☒	☐	A description of any assumptions or corrections, such as tests of normality and adjustment for multiple comparisons
☐	☒	A full description of the statistical parameters including central tendency (e.g. means) or other basic estimates (e.g. regression coefficient) AND variation (e.g. standard deviation) or associated estimates of uncertainty (e.g. confidence intervals)
☐	☒	For null hypothesis testing, the test statistic (e.g. *F*, *t*, *r*) with confidence intervals, effect sizes, degrees of freedom and *P* value noted<br>*Give P values as exact values whenever suitable.*
☐	☒	For Bayesian analysis, information on the choice of priors and Markov chain Monte Carlo settings
☒	☐	For hierarchical and complex designs, identification of the appropriate level for tests and full reporting of outcomes
☐	☒	Estimates of effect sizes (e.g. Cohen's *d*, Pearson's *r*), indicating how they were calculated

Our web collection on statistics for biologists contains articles on many of the points above.

Software and code

Policy information about availability of computer code

Data collection	No software was used for data collection
Data analysis	All software tools (and corresponding version numbers) used are provided in the Methods. These include SMNCopyNumberCaller (v1.1.1), CNVnator (v0.4.1), Quick-Mer2 (build 2021), Freebayes (v1.3.5), GATK HaplotypeCaller (v4.2.2), BWA, RTGtools (v3.12.1), NEAT (v3.0). The code for Parascopy is available at https://github.com/tprodanov/parascopy and we used v.1.2.6 of Parascopy (https://github.com/tprodanov/parascopy/archive/refs/tags/v1.2.6.zip) for the results and analysis presented in the paper.

For manuscripts utilizing custom algorithms or software that are central to the research but not yet described in published literature, software must be made available to editors and reviewers. We strongly encourage code deposition in a community repository (e.g. GitHub). See the Nature Portfolio guidelines for submitting code & software for further information.

Data

Policy information about availability of data

All manuscripts must include a data availability statement. This statement should provide the following information, where applicable:

- Accession codes, unique identifiers, or web links for publicly available datasets
- A description of any restrictions on data availability
- For clinical datasets or third party data, please ensure that the statement adheres to our policy

The analyses presented in this paper are based on the high-coverage whole genome sequencing data of 1000 Genomes Project samples that was generated at the New York Genome Center with funds provided by NHGRI Grant 3UM1HG008901-03S1. This sequencing data is available via ENA Study PRJEB31736 and PRJEB36890. The whole-genome sequence data for 90 Han Chinese samples is

available from ENA Study PRJEB11005. For this dataset, we used aligned reads downloaded from ftp.1000genomes.ac.uk/vol1/ftp/data_collections/han_chinese_high_coverage for analysis.
Source data are provided with this paper.

Field-specific reporting

Please select the one below that is the best fit for your research. If you are not sure, read the appropriate sections before making your selection.

☒ Life sciences ☐ Behavioural & social sciences ☐ Ecological, evolutionary & environmental sciences

For a reference copy of the document with all sections, see nature.com/documents/nr-reporting-summary-flat.pdf

Life sciences study design

All studies must disclose on these points even when the disclosure is negative.

Sample size	No sample size calculation was performed. The sample size for the WGS datasets were determined based on the size of the datasets that were publicly available.
Data exclusions	No data was excluded from the study
Replication	We assessed the replicability of the copy number estimation using WGS data on the same set of samples sequenced using two different library preparation protocols.
Randomization	This is not applicable since we did not have experimental groups.
Blinding	This is not applicable since we did not have experimental groups.

Reporting for specific materials, systems and methods

We require information from authors about some types of materials, experimental systems and methods used in many studies. Here, indicate whether each material, system or method listed is relevant to your study. If you are not sure if a list item applies to your research, read the appropriate section before selecting a response.

Materials & experimental systems

n/a	Involved in the study	
☒	☐	Antibodies
☒	☐	Eukaryotic cell lines
☒	☐	Palaeontology and archaeology
☒	☐	Animals and other organisms
☒	☐	Human research participants
☒	☐	Clinical data
☒	☐	Dual use research of concern

Methods

n/a	Involved in the study	
☒	☐	ChIP-seq
☒	☐	Flow cytometry
☒	☐	MRI-based neuroimaging
